# A Case of Trichorhinophalangeal Syndrome Caused by a Novel Heterozygous Nonsense Mutation in the *TRPS1* Gene

**DOI:** 10.1002/ccr3.70695

**Published:** 2025-07-31

**Authors:** Cailing E., Maaz Ahmed, Yu Wang, Jing Wang, Shixing Wu, Zudong Meng

**Affiliations:** ^1^ Department of Dermatology Renmin Hospital, Hubei University of Medicine Shiyan Hubei People's Republic of China

**Keywords:** case report, gene, nonsense mutation, trichorhinophalangeal syndrome, *TRPS1*

## Abstract

A 17‐year‐old male patient with a c.2065C>T heterozygous nonsense mutation in the *TRPS1* gene has sparse, soft hair; short thumbs and toes; misaligned teeth; and X‐ray findings of short distal thumb phalanges, depressed middle finger phalanges bases, and short toe proximal phalanges.

## 1 Introduction

Trichorhinophalangeal syndrome (*TRPS*) is a rare autosomal dominant genetic disorder caused by mutations in the *TRPS1* gene and is characterized by craniofacial abnormalities and skeletal deformities [1]. Clinical manifestations include sparse hair; distinctive facial features such as an absence of lateral eyebrows, fewer eyelashes, a pear‐shaped nose, a long philtrum, a thin upper lip and a smaller jawbone; and skeletal abnormalities such as short thumbs and toes, and conical epiphyses of the fingers and toes.

Based on genetic and clinical presentations, the disease can be divided into three types. Type I has typical clinical manifestations. Type II involves a contiguous gene syndrome with abnormalities in both the *TRPS1* and EXT1 genes [2], resulting in intellectual disability and multiple osteochondromas. Type III is a more severe form of Type I, characterized by more severe skeletal abnormalities, with significant short stature and brachydactyly. It has been suggested that *TRPS1* and *TRPS3* represent a spectrum of severity with some degree of genotype–phenotype correlation [3].

We report a case of trichorhinophalangeal syndrome and analyze the *TRPS1* gene mutation in the patient's family. This case report was prepared in accordance with the SCARE criteria [4].

## 2 Case Description

### 2.1 Case History/Examination

**FIGURE 1:**
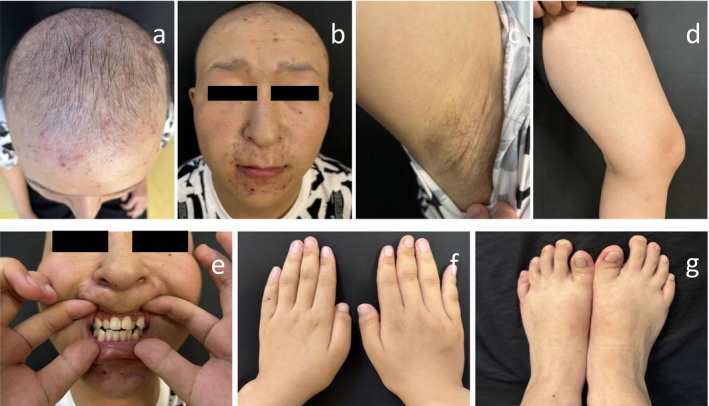
Clinical manifestations of the proband. (a) Diffuse sparse hair with varying thickness and length, soft and yellowish color; (b) special facial features include sparse outer eyebrows; fewer eyelashes; a bulbous nose; a medium length, a thinner upper lip; and multiple erythema, follicular papules, and pustules on the face, mouth area, and scalp; (c, d) less hair on the whole body, and sparse hair on the armpits and limbs; (e) uneven arrangement of teeth (supernumerary teeth, crowding); (f, g) normal toenails. The thumb and toe are noticeably shorter.

The patient is a 17‐year‐old male who presented with “sparse and soft hair for 17 years.” He was born with sparse hair all over his head, which was yellow, soft, and thin, and could grow to approximately 3 cm before falling out. Over the past 4 years, he has had recurrent scattered papules and pustules on the anterior face, which have been treated with oral Chinese medicine, topical sulfur ointment, and fusidic acid cream, with symptoms recurring. He had no history of other systemic diseases and denied drug allergies. His parents are healthy, not consanguineous, and deny a family history of hair loss or genetic diseases. His 20‐year‐old sister was normal in terms of growth, development, and hair. Specialized examination (Figure 1) revealed that the hair was diffusely sparse, uneven in thickness and length, and soft and yellowish in color. The lateral eyebrows were sparse, the eyelashes were few, the nose was globular, the philtrum was long, and the upper lip was thin. There was sparse vellus hair on the body, and the axillary and limb hair were sparse, with no abnormalities in the nails of the fingers or toes. The thumbs and big toes were significantly shorter. Dental misalignment (including supernumerary teeth and crowding) was noted. There was no congenital cataract, and growth, development, intelligence, and peers were not significantly different, with a height of 175 cm. There were multiple erythaematous patches, follicular papules, and pustules on the face, perioral area, and scalp. He would sweat normally.

### 2.2 Differential Diagnosis, Investigations, and Treatment

**FIGURE 2:**
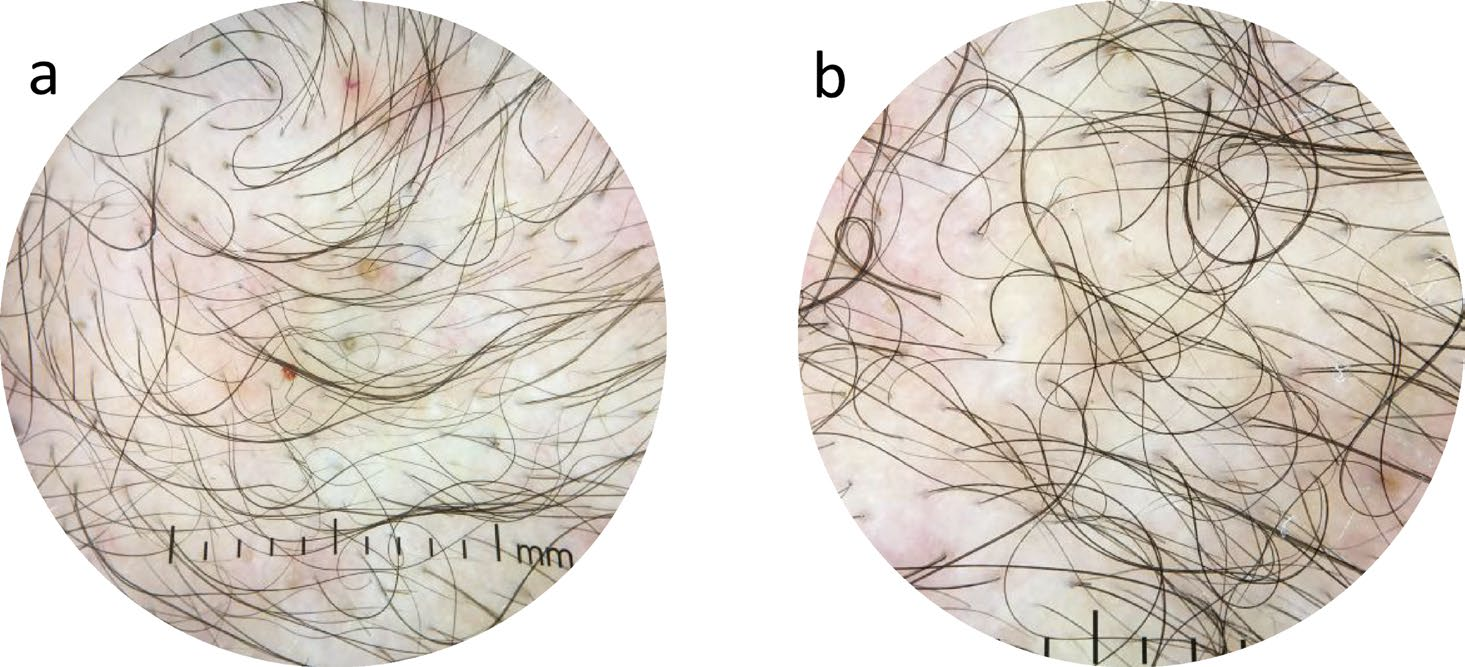
Dermoscopy detection results. (a, b) The proportion of single hair follicle units in different parts of the scalp have significantly increased, the diameter of the hair shaft has become thinner, and broken hair can be seen, with multiple follicular papules and pustules.

**FIGURE 3:**
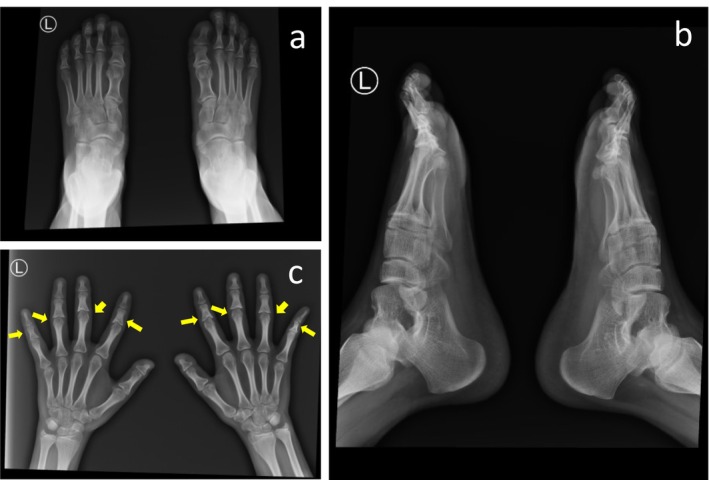
X‐ray examination. (a) Bilateral X‐ray anteroposterior view: short proximal phalanges of the big toe of both feet; (b) Bilateral X‐ray lateral view: the both feet showing short proximal phalanges of the big toe; (c) Bilateral X‐ray: brachymetacarpia (short metacarpal bones), multiple cone‐shaped epiphyses, shorter distal phalanx of thumb, the base of the middle phalanges of the 2nd to 5th fingers is depressed, and the adjacent phalanges become pointed, presenting as cone‐shaped epiphyses (indicated by the arrow).

The laboratory test results revealed no significant abnormalities in serum testosterone, total cholesterol, or thyroid function, with a mild decrease in 25‐hydroxyvitamin D3 (23.73 ng/mL). Dermoscopy detection (Figure 2) revealed a significant increase in the proportion of single hair follicular units, with thinner hair shafts, visible broken hairs, and multiple follicular papules and pustules. X‐ray examination (Figure 3) revealed that the distal phalanges of both thumbs were short, the middle phalanges of the second to fifth fingers had a concave base with the adjacent finger head becoming pointed, the proximal phalanges of both large toes were short, and no other significant abnormalities were observed.

**FIGURE 4:**
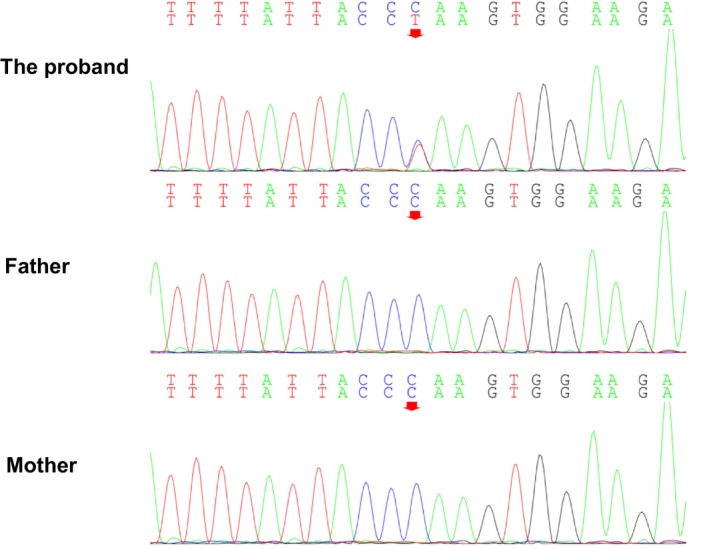
Family Sanger sequencing results. There is a heterozygous nonsense mutation on exon 4 of the TRPS1 gene in the proband, c.2065C>T, p.Gln689*. The proband's parents have no gene mutation at this locus.

After informing the patient's family about the voluntary nature and confidentiality of the study and obtaining informed consent, peripheral blood samples were collected from the patient and two other family members for genetic sequencing analysis and related laboratory tests. Panel targeted sequencing revealed (Figure 4) a mutation in the fourth exon of the *TRPS1* (NM_014112) gene on the patient's chromosome 8 at the base position 116616131, with a c.2065C>T mutation. This variant has not been found in healthy individuals or genetic databases. The variant frequency is not recorded in databases such as gnomAD_exome, HGMD, 1000 Genomes, and ExAC. According to the American College of Medical Genetics and Genomics (ACMG) guidelines, novel mutations can be categorized into one or more of five classifications as follows: PVS1 (very strong pathogenic evidence): loss‐of‐function variants (e.g., nonsense mutations, frameshift mutations, etc.); PS2 (strong pathogenic evidence): de novo (new) variant, with no family history; PS4 (supporting pathogenic evidence): the variant is significantly more frequent in affected individuals than in controls; PM2 (supporting pathogenic evidence): the variant is not found in the general population in public databases; and PP4 (supporting pathogenic evidence): the clinical features or family history of the variant carrier are highly consistent with a single‐gene disorder.

According to the ACMG guidelines, which combine the above variation evidence and the pathogenicity level of evidence classification, this variant is classified into two types of strong pathogenic evidence and one type of pathogenic evidence: 2 PS2_Strong + 2 PM2_Supporting. This means that it may be a pathogenic variant. MutationTaster predicts the result as “likely pathogenic,” and the phenotype of the proband's hair, eyebrows, skeletal changes, and facial features is consistent with this monogenic disease. When the patient's clinical characteristics, gene mutation detection results, and protein function prediction results are combined, the diagnosis of *TRPS* is clear. No reports have been found in various databases, indicating that this gene mutation is new. The patient has no intellectual abnormalities and can live a normal life no different from an ordinary person, so there is no special treatment. Only symptomatic treatment is used for folliculitis.

### 2.3 Outcome and Follow‐Up

At present, there is no mature gene therapy plan applied to the clinical treatment of *TRPS1* gene disease. We closely monitor the patient's growth and development, endocrine function assessment, bone and joint condition, as well as genetic counseling and family screening during follow‐up.

## 3 Discussion

*TRPS* is a rare autosomal dominant hereditary disease characterized by high penetrance and a wide range of phenotypic variability. *TRPS* was first reported by Giedion in 1966 [5], and many patients with *TRPS* may remain undiagnosed, making it impossible to estimate the prevalence of *TRPS* without bias in the population. Type I *TRPS* (MIM 190350) is characterized by typical clinical manifestations, including sparse hair and craniofacial and skeletal abnormalities [6, 7]. Patients with *TRPS* Type I generally do not have intellectual disability or significant short stature and are usually caused by nonsense mutations in the *TRPS1* gene. *TRPS* Type II, in addition to typical manifestations, is characterized by intellectual disability, multiple ossifications, and multiple exostotic osteochondromas [8]. *TRPS* Type III is a more severe form of *TRPS* Type I, and data suggest that *TRPS III* is at the severe end of the *TRPS* spectrum, most commonly caused by a specific class of mutations in the *TRPS1* gene [9].

*TRPS* is an uncommon condition, contributing to its low recognition among healthcare providers outside specialized fields. In some cases, the syndrome's mild or variable symptoms can result in prolonged periods between initial signs and a confirmed diagnosis, as observed in individuals with delayed identification following skeletal manifestations. Clinical evaluation and imaging studies form the cornerstone of *TRPS* diagnosis. Imaging of the hands and feet frequently reveals distinctive features, such as abnormally short metacarpal or metatarsal bones and cone‐shaped growth plates in the phalanges, which aid in strengthening diagnostic suspicions. A conclusive diagnosis is achieved through molecular testing that detects disease‐causing mutations in the *TRPS1* gene. In clinical practice, *TRPS* needs to be differentiated from other diseases with similar sparse hair or skeletal abnormalities. For example, if hair loss is the main manifestation, it needs to be differentiated from androgenetic alopecia in clinical practice, and genetic sequencing may be necessary for a definitive diagnosis [10]. In addition, it is necessary to distinguish it from other genetic syndromes such as Cornelia de Lange syndrome [1] with sparse hair and skeletal abnormalities, which are often accompanied by intellectual disabilities and growth retardation. Studies have revealed the skeletal and dental abnormalities of *TRPS1* [6], and described characteristic X‐ray features such as cone‐shaped epiphyses in fingers and toes [7]. These skeletal abnormalities are important diagnostic criteria for *TRPS* and help differentiate it from other genetic syndromes.

The genotype–phenotype correlation in *TRPS* Type I has always been a controversial issue. Studies have revealed significant variation within and between families with the same *TRPS* Type I pathogenic variant [11]. *TRPS* Type I disease is autosomal dominant, and a patient's child has a 50% chance of inheriting the *TRPS1* pathogenic variant. For many, *TRPS* may still be challenging and undiagnosed [12]. Once a *TRPS* genetic variant is identified in a patient's family members, prenatal testing or genetic diagnosis becomes necessary [13]. The clinical trajectory of patients varies and involves pediatrics, dermatology, orthopedics, clinical genetics, and/or dentistry, emphasizing the importance of close multidisciplinary cooperation for early diagnosis of *TRPS* and ensuring appropriate and timely patient care and counseling [14]. The *TRPS1* gene produces a zinc finger transcription factor critical for maintaining bone balance by controlling mineralization around cartilage, cartilage cell growth, and programmed cell death. Notably, clinical features linked to *TRPS1* dysfunction resemble those observed in individuals with low zinc levels, as zinc deficiency can impair zinc‐dependent proteins, potentially exacerbating the condition by worsening clinical manifestations [15]. This patient's *TRPS1* gene has a heterozygous nonsense mutation, c.2065C>T, p.Gln689*, which changes an evolutionarily highly conserved amino acid residue, leading to premature termination of polypeptide chain synthesis, premature truncation of the *TRPS1* protein, and loss of its functionality. This functional loss mutation may be the main cause of the patient's typical clinical manifestations, such as sparse hair, short finger deformities, cone‐shaped epiphyses. However, the relationship between *TRPS1* genotype and phenotype is not entirely consistent, and even among carriers of the same gene mutation, there may be significant differences in phenotype. This may be related to the regulation of gene expression, the role of modifying genes, and the influence of environmental factors [9, 12, 16].

In recent years, some large‐scale cohort studies have further revealed the clinical characteristics and gene mutation spectrum of *TRPS*. For example, Herlin et al. [14] found in their study of 15 patients with *TRPS* that the type and location of *TRPS1* gene mutations have a significant impact on phenotype, but the severity of phenotype still varies among individuals. The c.2065C>T mutation carried by the patient in this case has not been reported in previous studies, suggesting that the mutation spectrum of the *TRPS1* gene may be more complex than currently known. In addition, the study emphasizes the importance of interdisciplinary collaboration in the diagnosis and management of *TRPS*, which is consistent with the diagnosis and treatment process of the patient in this case.

## 4 Conclusion

In summary, a mutation in the 4th exon of the *TRPS1* (NM_014112) gene on chromosome 8 was discovered for the first time, with a c.2065C>T mutation, meaning the base pair at position 2065 was mutated from C to T. This mutation was not found in the parents, indicating a de novo mutation. Our research on *TRPS* mutations has broadened the mutation and phenotype spectrum of *TRPS*. Our results reveal the importance of *TRPS1* molecular analysis in improving the clinical diagnosis of TRPS.

## Author Contributions

**Cailing E.:** writing – original draft. **Maaz Ahmed:** resources. **Yu Wang:** writing – review and editing. **Jing Wang:** formal analysis. **Shixing Wu:** visualization. **Zudong Meng:** conceptualization, methodology.

## Ethics Statement

The case report was approved by the Ethics Review Committee of the Renmin Hospital, Hubei University of Medicine.

## Consent

Due to the patient being a minor, this case report has obtained written informed consent from the patient's father for publication.

## Conflicts of Interest

The authors declare no conflicts of interest.

## Supporting information


Data S1:

## Data Availability

The authors confirm that the data supporting the findings of this study are available within the article and its Supporting Information.
