# Supplementary material for: A Case of Trichorhinophalangeal Syndrome Caused by a Novel Heterozygous Nonsense Mutation in the TRPS1 Gene
Source: Clin Case Rep. 2025 Jul 31;13(8):e70695. doi: 10.1002/ccr3.70695 (PMC12313832; doi:10.1002/ccr3.70695)
Supplement: Supplementary file 1 — Data S1: [file CCR3-13-e70695-s001.docx]

**I. Materials and methods**

**1. Subject of the study**

A patient with suspected Tricho-rhino-phalangeal syndrome disease attending Shiyan People's Hospital in July 2024 was selected for the study.

**2. Research methodology**

**2.1 Collection of blood samples**

After the subjects signed the consent form, 2 ml of venous blood was collected and placed in an anticoagulant tube containing ethylenediamine tetraacetic acid (EDTA), and sent to Beijing Kangxu Medical Laboratory for whole exome testing.

**2.2 DNA extraction and concentration determination**

2.2.1 DNA extraction

The genomic DNA of the blood samples was extracted using Qiagen Flexi Gene DNA Kit (No.51206, Qiagen, Germany) and stored at-20℃. The DNA extraction process was carried out according to the instruction of the kit.

2.2.1.1 Materials and instruments

Blood samples, 100% isopropyl alcohol, 70% ethanol, 1.5 ml microtubes, 2 ml centrifuge tubes and tips, vortex oscillator, centrifuge, water bath, ice crusher.

2.2.1.2 DNA Extraction Process

(1) Add 5 ml of FG1 Buffer to a 15 ml centrifuge tube, then incorporate 200 μl of whole blood. Invert the tube back and forth 5 times to ensure thorough mixing (if the blood sample is resistant to dissolution, use an SBP mixer for 10 minutes).

(2) Centrifuge at 2,000 × g for 3 min;

(3) Pour off the supernatant and invert the centrifuge tube onto clean blotting paper for 2 min to aspirate the plasma as cleanly as possible, being sure to take care that the particles do not slip off;

(4) Add 1.5 ml of FG2/QIAGEN Protease Buffer, cover the centrifuge tube, and vortex rapidly to mix until the pellet is completely dissolved;

(5) Centrifuge the tube at low speed for 3-5s and incubate at 65℃ for 5min, (the color changes from red to olive green);

(6) Add 1.5 ml of 100% isopropanol and mix upside down with the SBP mixer until a distinct line or a clump of DNA;

(7) Centrifuge at 2,000 × g for 5 min;

(8) Pour on the supernatant and briefly turn the centrifuge tube on clean blotting paper;

(9) Add 100μl 70%ethanol mixture and vortex to mix for five seconds;

(10) Centrifuge at 2,000 × g for 3 min;

(11) Pour on clean blotting paper (≥5 min) of the supernatant and invert it;

(12) Repeat steps 9.10.11 once or twice, as determined by the color of the washed liquid and precipitate;

(13) Air-dry the DNA pellet until all liquid has evaporated (at least 5 min);

(14) Add 200μl of FG3 Buffer, vortex at low speed for 5s, and incubate at 65℃ for 1h;

(15) After quantification by Nanodrop, store at -20℃ for backup;

2.2.2 Determination of DNA concentration

Using a nanodrop 2000 Ultra-Micro Spectrophotometer (Thermo Fisher Scientific, USA), the DNA content was ascertained in order to guarantee that the OD 260/280 of genomic DNA in the samples was between 1.8 and 2.0, the concentration of 500 ng/μl; the total amount of genome was more than 3.5 μg, and the concentration of DNA was>100 ng/μl. The genomic integrity of the samples should be detected by agarose electrophoresis.

**2.3 Selection of target genes and probe design**

With reference to OMIM and HGMD databases, we selected genes related to various types of genetic diseases, and utilized Agilent (Agilent, Inc., USA) SureDesign online design tool to design target capture probes for exons and flanking sequences (+-10bp) of the target genes, and customized special capture kits for the target genes.

**2.4 Genomic DNA library construction**

The SureSelect Target Enrichment System Target Sequence Enrichment Kit (the item number should be the same as the item number of the probe that came in at that time, Agilent Company, USA) was used to prepare target gene capture libraries for the DNA samples of the suspected TRPS patients, and the operation steps were carried out strictly according to the instructions.

(1) The DNA samples were fragmented using a Covaris Ultrasonic DNA Crusher and SonoLab software version 7.0 to obtain 100-500bp DNA fragments.

(2) DNA sample fragments were purified using AMPure XP magnetic beads.

(3) Evaluate the quality of the DNA samples using the 2100 Bioanalyzer instrument and the DNA 1000 kit. (Optional)

(4) Repeat DNA fragment ends with the Yes Select XT Library Prep Kit ILM. Create the proper amount of master mix (Table 1) that is finished in ice and mix on a vortex shaker. Add 52 μl of master mix to the purified DNA fragments on the PCR plate and mix well by blowing with a lance tip. Step 1: insert the PCR plate into the PCR equipment and then proceed with the application in Table 2.

**Table 1 Preparation of end-repair master mix**

| reagents | 1 reaction dose |
| --- | --- |
| Nuclease-free water | 35.2 μl |
| 10× End Repair Buffer | 10 μl |
| dNTP Mix | 1.6 μl |
| T4 DNA Polymerase | 1 μl |
| Klenow DNA Polymerase | 2 μl |
| T4 Polynucleotide Kinase | 2.2 μl |
| in sum | 52 μl |

**Table 2 End-repair PCR program**

| move | temp | timing |
| --- | --- | --- |
| 1 | 20°C | 30 min |
| 2 | 4°C | save (a file etc) (computing) |

1. DNA fragments were purified using AMPure XP magnetic beads.
2. Adenylate the 3'end of the DNA fragment using the SureSelect XT Library Prep Kit ILM. Prepare an appropriate amount of adenylation master mix (Table 3) on ice and mix on a vortex shaker. Add 20 μl of adenylation master mix to the end-repaired purified DNA fragments and mix by blowing with the head of the gun. Take the sample from the PCR equipment and use the software listed in Table 4.

**Table 3 Preparation of adenylated master mixes**

| reagents | 1 reaction dose |
| --- | --- |
| Nuclease-free water | 11 μl |
| 10× Klenow Polymerase Buffer | 5 μl |
| dATP | 1 μl |
| Exo(-) Klenow | 3 μl |
| in sum | 20 μl |

**Table 4 dA-Tailing PCR program**

| move | temp | timing |
| --- | --- | --- |
| 1 | 37 °C | 30 min |
| 2 | 4°C | save (a file etc) |

1. DNA fragments were purified using AMPure XP magnetic beads.

(8) Use the SureSelect XT Library Prep Kit ILM to attach a junction paired with the end of the DNA fragment. Prepare an appropriate amount of ligation master mix (Table 5) on ice and mix on a vortex shaker. Add 37 μl of ligation master mix to the end-adenylated, purified DNA fragments on the PCR plate and mix by pipetting with a lance tip.Insert the PCR plate into the PCR machine and adhere to the program outlined in Table 6.

**Table 5 Connected master mix preparation**

| reagents | 1 reaction dose |
| --- | --- |
| Nuclease-free water | 15.5 μl |
| 5× T4 DNA Ligase Buffer | 10 μl |
| Sure Select Adaptor Oligo Mix | 10 μl |
| T4 DNA Ligase | 1.5 μl |
| in sum | 37 μl |

**Table 6 Connected PCR programs**

| move | temp | timing |
| --- | --- | --- |
| 1 | 20 °C | 15min |
| 2 | 4°C | save (a file etc) (computing) |

(9) DNA fragments were purified using AMPure XP magnetic beads.

(10) AMAGES THE DNALibrarywiththelonging. Using ice, the right amount of master mix was made (Table 7). Mix on a vortex shaker. 35 μl of this master mix is mixed with 15 μl of purified DNA library. Mix 35 μl of this master mix with 15 μl of purified DNA library.1 DNA library sample is added to one well of the PCR plate. Use a lance tip to agitate the mixture. Next, insert the PCR plate into the PCR machine and adhere to the protocol outlined in Table 8.

**Table 7 SureSelect Pre-Capture PCR Reaction Mix**

| reagents | 1 reaction dose |
| --- | --- |
| Nuclease-free water | 21 μl |
| SureSelect Primer | 1.25 μl |
| SureSelect ILM Indexing Pre-Capture PCR PCR  Reverse Primer | 1.25 μl |
| 5× Herculase II Reaction Buffer | 10 μl |
| 100 mM dNTP Mix | 0.5 μl |
| Herculase II Fusion DNA Polymerase | 1 μl |
| in sum | 35 μl |

**Table 8 Pre-capture PCR program**

| move | cycle number (math.) | temp | timing |
| --- | --- | --- | --- |
| 1 | 1 | 98 °C | 2 min |
| 2 | 4-6 | 98°C  65°C  72°C | 30 s  30 s  1 min |
| 3 | 1 | 72°C | 10 min |
| 4 | 1 | 4°C | save (a file etc) |

1. Using AMPure XP magnetic beads，purify the amplified DNA library.
2. The quality and amount of the DNA libraries were measured using a 2100 Bioanalyzer equipment with DNA 1000 kit.

**2.5 Hybridization and capture**

Prepared genomic DNA libraries are hybridized to target-specific capture libraries. After hybridization, target molecules are captured using streptavidin-labeled magnetic beads. Each DNA library sample must be hybridized and captured individually before the indexing tag is added by PCR. The ratio of capture libraries to genomic DNA libraries is critical for successful capture.

2.5.1 Hybridization of DNA samples to capture libraries

The Sure Select XT Reagent Kit is used to hybridize prepared genomic DNA libraries to target-specific capture libraries. Table 9 lists the reagents that were used for hybridization. If the concentration of DNA in the prepared DNA library is greater than 221 ng/μl, prepare 3.4 μl of each library. If the concentration of DNA in the prepared DNA library is less than 221 ng/μl, concentrate the sample DNA using a vacuum concentrator at 45°C, re-dilute to a concentration of 221 ng/μl, mix on a vortex oscillator, and centrifuge for 1 minute.Transfer each 3.4μl genomic DNA library sample to each well of a 96-well plate, seal the wells and store on ice. Create a hybridization buffer (Table10) at room temperature and at the same time that it was kept at that temperature. Examine the precipitation at 65℃ for five minutes in order to incubate the hybridization buffer. Preparation of Sure Select Block Mix (Table11) and keep on ice for later use.

**Table 9 Reagents for hybridization**

| Kit composition | save |
| --- | --- |
| Sure Select Hyb 1 | Sure Select Target Enrichment-Box 1, RT |
| Sure Select Hyb 2 | Sure Select Target Enrichment-Box 1, RT |
| Sure Select Hyb 3  Reverse Primer | Sure Select Target Enrichment Kit ILM  Indexing Hyb Module Box 2, -20°C |
| Sure Select Hyb 4 | Sure Select Target Enrichment-Box 1, RT |
| Sure Select Indexing Block 1 | Sure Select Target Enrichment Kit ILM  Indexing Hyb Module Box 2, -20°C |
| Sure Select Block 2 | Sure Select Target Enrichment Kit ILM  Indexing Hyb Module Box 2, -20°C |
| Sure Select ILM Indexing Block 3 | Sure Select Target Enrichment Kit ILM  Indexing Hyb Module Box 2, -20°C |
| Sure Select RNase Block | Sure Select Target Enrichment Kit ILM  Indexing Hyb Module Box 2, -20°C |
| Capture Library | -80°C |

Add 5.6 μl of Sure Select Block Mix to each genomic DNA library sample and mix well with a lance tip. Table 12 reports that the PCR plate in the PCR equipment is located in Seal the wells, which then runs the instrument in accordance with the program.

**Table 10 Preparation of hybridization buffer**

| reagents | 1 reaction dose |
| --- | --- |
| Sure Select Hyb 1 | 6.63 μl |
| Sure Select Hyb 2 | 0.27 μl |
| Sure Select Hyb 3 | 2.65 μl |
| Sure Select Hyb 4 | 3.45 μl |
| in sum | 13 μl |

**Table 11 Preparation of SureSelect Block Mix**

| reagents | 1 reaction dose |
| --- | --- |
| Sure Select Indexing Block 1 | 2.5 μl |
| Sure Select Block 2 | 2.5 μl |
| Sure Select ILM Indexing Block 3 | 0.6 μl |
| in sum | 5.6 μl |

**Table 12 PRC procedure for DNA + Block Mix before hybridization**

| move | temp | timing |
| --- | --- | --- |
| 1 | 95 °C | 5min |
| 2 | 65 °C | Save (at least 5min) |

Depending on the size of the libraries captured in the experiment, the appropriate dilutions of Sure Select RNase Block were prepared (Table13), keep on ice and set aside.

**Table 13 Preparation of RNase Block dilutions**

| Capture library size | RNase Block dilution (RNase Block: water) | RNase for hybridization  Block Dilution Volume |
| --- | --- | --- |
| ≥3.0 Mb | 25% (1:3) | 2 μl |
| <3.0 Mb | 10% (1:9) | 5 μl |

Prepare the Capture Library Hybridization Mix based on either Table 14 (for capture libraries ≥ 3.0 Mb) or Table 15 (for capture libraries<3.0 Mb) depending on the sizes of the capture libraries in your experiment, vortex the mixture, and store it at room temperature.

**Table 14 Preparation of the capture library with 3.0 Mb of capture library**

| reagents | 1 reaction dose |
| --- | --- |
| Hybridization Buffer mixture | 13 μl |
| 25% RNase Block solution | 2 μl |
| Capture library ≥ 3.0 Mb | 5 μl |
| in sum | 20 μl |

**Table 15 Preparation of Capture Library Hybridization Mix(capture library<3.0 Mb)**

| reagents | 1 reaction dose |
| --- | --- |
| Hybridization Buffer mixture | 13 μl |
| 10% RNase Block solution | 5 μl |
| Capture library <3.0 Mb | 2 μl |
| in sum | 20 μl |

Maintain the temperature of the Genomic DNA Library + Block Mix plate at 65 °C while adding 20 μl of Capture Library Hybridization Mix to each well, and mix by blowing 8-10 times with the tip of the gun. Depending on how much evaporation happens during the PCR reaction, the volume of the hybridization reaction solution is roughly 27-29 μl. Seal the wells with the PlateLoc Thermal Microplate Sealer, making sure that the plate wells are completely capped.at the temperature of 65 °C, incubate the hybridization mixture at 65 ° C for 16 or 24 h, and then use a thermal capping temperature of 105 °C.

2.5.2 Preparation of streptavidin-labeled magnetic beads

Pre-warm Yes Select Wash Buffer 2 in a circulating water bath at 65 °C, resuspend the Dynabeads MyOne Streptavidin T1 beads on a vortex shaker, and let stand for storage.In each well of the PCR plate, incorporate 50 μl of the resuspended magnetic beads into every hybridization sample.

Wash magnetic beads: Apply 200μl of Sure Select binding Buffer, then combine it by means of a gun pipetting until the beads are totally resuspended. Place the plate in a magnetic separator until the solution is clear and remove the supernatant. Repeated the preceding procedures. Use 200μl of SureSelect Binding Buffer to respend the beads.

2.5.3 Capture of hybridized DNA with streptavidin-labeled magnetic beads

Evaluate and record the volume of hybridization solution after 24 hours of incubation. Maintain the hybridization temperature at 65 °C while using a multichannel pipette to transfer the full volume of each hybridization mixture (~25-29 μl) to plate wells containing 200 μl of washed Streptavidin-labeled magnetic beads, and slowly mix by pipetting with the tip of a gun until the beads are completely resuspended. After being placed in an empty lid, the capture plate was placed in a 96-well plate mixer, which was then vigorously mixed at room temperature (1400-1800 rpm) for 30 minutes.Make sure the sample is thoroughly mixed within the wells. Centrifuge the plate briefly in a centrifuge. Place the plate in a magnetic separator to collect the beads. When the solution is clear, remove the supernatant. After being placed inside 200 µl of Sure Select Wash Buffer 1, the beads were mixed with a suction to the diameter of the lance until they were completely resuspended. Samples are kept at room temperature for 15 minutes in order to incubate. A centrifuge of the sample in a short amount of time. Before the supernatant is removed, place the well plate in a magnetic separator and wait for the solution to be cleared.

Clean the beads using Sure Select Wash Buffer. Respend 200 µl of preheated Wash Buffer at 65 °C, mix it by pipetting the tip of a gun until the beads were entirely resuspended. After incubating the plate in the PCR equipment at 65 °C for ten minutes, cover the wells. Insert the plate into a magnetic separator and allow the solution to clarify before carefully removing the supernatant. Repeat this process two more times. Make sure to remove all eluent in the last wash. Add 30 μl of nuclease-free water to each sample well and resuspend the magnetic beads by blowing on them with a lance tip. Keep the samples on ice for backup.

**2.6 Pre-sequencing sample processing**

PCR amplification of Sure Select-enriched DNA libraries was performed using indexing primers with 8 bp index A01-H12 to amplify the captured libraries. Using AMPure XP beads, purify the amplified captured library.Assess the amount and quality of DNA in the indexed library. DNA quality and quantity were assessed using a 2100 Bioanalyzer instrument (Agilent, USA) and a High Sensitivity DNA kit. Prepare sequencing samples.

**2.7 Sequencing**

Sequencing was performed on the T7 platform according to the concentration of the captured library DNA sample and the sequencing depth, and the operation steps were carried out in strict accordance with the instructions given by the instrument manufacturer. The amplified products of T7 platform were sequenced bipartite, and Fastaq format data were obtained. Sequencing of both ends of the amplified product of the T7 platform, applying the principle of sequencing while synthesizing. Four fluorescent markers are incorporated into the modified DNA polymerase and dNTP. Because of the chemically cleavable part of the 3'hydroxyl terminus, which permits the inclusion of only one base per loop, these nucleotides are "reversible terminators". At this point, the surface of the reaction plate is scanned with a laser to read the kind sofnucleotides that have been polymerized in the first round of reactions for each cross plate sequence. Afterwards, these bases are chemically cut to restore the 3' hydroxyl end stickiness and the polymerization of the second nucleotide continues. This continues until each template sequence is completely polymerized as a double strand. In this way, the results of the fluorescence signals collected in each round are counted to know that each template Sequence of DNA fragments.

**2.8 Data analysis (bio informatics analysis)**

2.8.1 Data splitting

Use bcl2fastq (t 2) to transform the bcl format of the sequencing data into the fastq format.

2.8.2 Comparison and quality control

Sequencing reads were compared to the human reference genome (hg19 version) using the tool BWA (v0.7.15). The results were quality controlled by a variety of metrics, including global alignment depth, interval alignment depth, interval coverage, and locus alignment quality.

2.8.3 Analysis of variations

GATK (v3.6) was used to find the comparison results in the form of SNV (single nucleotide variants) and in Del (small insertion deletion variants), which were then converted into bam format. SNVs (single nucleotide variants) and In Del (small insertion deletion variants) were detected using CODEX, XHMM (v1.0) and KSCNV (developed by Kangxu) was examined for possible CNV (copy number variation).

2.8.4 Annotation analysis

a) ANNOVAR (v2016-02-01) was used to annotate the positions of the variations in the genes and transcripts.

b) Perform gene-related annotations involving RefSeq, Ensembl, UCSC and other databases.

c) The frequency of annotated variants in the population, including 1000G, dbSNP, ESP6500, ExAC, etc. One database of single nucleotide polymorphisms in the Thousand Genomes Project is called 1000G. Among them, 1000G is the frequency of variants in the Thousand Genomes Project, which includes two versions, V1 and V3. dbSNP is a single nucleotide polymorphism database, ESP6500, and ExAC are large-scale population-wide exome databases.

d) Using the PolyPhen2, SIFT, and Mutation Taster tools, protein damage analysis was carried out. These three tools are used to assess the extent of protein damage caused by variants. Phenoplastic, or benign; SIFT classifies results as either Tolerated or Deleterious; and MutationTaster classifies findings into polymorphism, which is automatic, disease-causing, and disease-causing; specifically, PolyPhen2 divides results into Benign, Possibly Damaging, and Probably Damaging.

e) Disease-related annotations, such as the ClinVar, HGMD, OMIM, and so forth. The OMIM database will annotate the official name of the gene, the English name of the disease, the Chinese name of the disease, the mode of inheritance and other information. HGMD annotates references to variants associated with disease. ClinVar provides information onthedisease-causing effects of variants that have been reported by other laboratories.f)Other analyses, e.g., GERP + + conservativeness analysis.

2.8.5 Filtration

The annotation results are filtered in terms of variant location, variant type, variant frequency, and characterization of the variant locus in the database to retain variants that may be associated with disease.

2.8.6 Annotation ACMG variant grading

The ACMG Variant Classification is a standard and guideline for interpreting genetic variants published by the American College of Medical Genetics and Genomics (ACMG) in conjunction with the Academy of Molecular Pathology (AMP), which categorizes variants into five classes, namely, pathogenic, probably pathogenic, of uncertain significance, probably benign, and benign. Pathogenicity evidence is addressed in PVS1, PS1-4, PM1-6, and PP1-5; non-pathogenicity evidence is addressed in BA1, and BS1-4 and BP1-6.

2.8.7 Clinical analysis

Separate clinical analyses were performed on different samples, including clinical symptom matching, genetic pattern matching, etc.

**2.9 First-generation sequencing validation**

Generation validation was performed for the loci to be validated in this sample and for the loci in its family samples. The gene sequences of the above mutated loci were obtained from the human genome database GenBank, and primers were designed and synthesized at the primer design website Primer Z (http://genepipe.ncgm.sinica.edu.tw/primerz/primerz4.do). Following the PCR amplification of the mutant loci, one-generation sequencing was conducted, and the resulting sequences werecompared to the earlier sequences to eliminate any false-positive loci identified in the second-generation sequencing. The specific process was as follows:

2.9.1 Genomic DNA extraction

We extracted genomic DNA from blood samples by the Gene DNA kit method and performed quality control of genomic DNA. For genomic QC, the instrument used is: NanoDrop2000, the concentration of genome we need is 50-100ng/ul, the OD (260/280) value is 1.8-2.0, the total amount of genome depends on the number of gene reactions, and the amount of DNA needed for each reaction is 50 ng.

2.9.2 Primer design and amplification analysis

Primers were created for the single genes examined using Primer Z. Primers were synthesized, diluted and then amplified by PCR according to the primer conditions using Goldstar Taq mix and an ABI PCR amplifier. The PCR products were then electrophoresed for 1% agarose gel electrophoresis in order to identify, purify, and recover the products. The retrieved items were then subjected to primary sequencing (ABI 3730 Primary Sequencer), and the results of the primary sequencing were interpreted and analyzed to exclude false-positive sites from the secondary sequencing.

**II. Results**

The patient was a 17-year-old male with thin, fine, and limp hair. The patient was born with sparse hair on the whole head, which was yellow, soft and fine, and grew to about 3cm with age, then fell off. For the past four years, he has experienced recurrent scattered papules and pustules on the front of his head, having undergone treatment with oral traditional Chinese medicine, topical sulfur ointment, and fusidic acid cream, yet his symptoms persist.There was no history of other systemic illnesses and drug sensitization was denied. Parents are healthy, not consanguineous marriage, deny alopecia and family history of hereditary diseases, his sister is 20 years old, growth and development and hair is normal. Specialty (Figure 1): diffuse thinning hair, thickness, length, soft, yellowish color, sparse outer eyebrows, fewer eyelashes, bulbous nose, long middle, thin upper lip. The whole body has less cui hair, sparse axillary hair and limb hair, pubic hair is not abnormal, finger and toe nails are normal. Thumb, lesser toes obviously shorter. Teeth are not aligned (multiple teeth, crowding). There was no congenital cataract, and no significant differences were observed in growth, development, or intelligence compared to peers without any issues. Multiple erythematous spots, follicular papules and pustules were seen on the face, perioral area and scalp. Sweating was normal.

Blood test results suggested: serum testosterone, total cholesterol, and methyl function were not significantly abnormal, and 25-hydroxyvitamin D3 was mildly decreased (23.73ng/ml). Trichoscopy (Figure 2):the proportion of follicular units of a single hair increased significantly, the diameter of the hair shaft was thinned, hair breakage was seen, and there were multiple follicular papules and pustules. X-ray examination (Figure 3): the distal phalanges of the thumbs of both hands were short, the bases of the middle phalanges of the 2nd-5th fingers of both hands were sunken, and the heads of the neighboring phalanges were pointed, and the proximal phalanges of the lesser toes of both feet were short; the rest of the examination did not show any significant abnormality.

After signing the informed consent, the patient was told that the study was voluntary and private; following the signing of the informed permission, the patient's peripheral blood was taken from the patient and the other two members of the family; and thereafter, the gene sequencing analysis and related laboratory testing were performed. Panel targeted sequencing (Figure 4) revealed the presence of the exon 4 of the *TRPS I* (NM_014112) gene on chromosome 8, at the base site of 116616131, which suggests that this is a c.2065C>T mutation. A mutation in exon 4 of the patient's chromosome 8 at the base locus 116616131 resulted in the c.2065C>T mutation, i.e., the base pair 2065 was mutated from a C to a T. This was the result of a denovomutation in which there was neither of the patient's parents; this was a new mutation which causes a change from Gln to the stop codon, which was the amino acid 689 encoded by the gene being changed from Gln (chr8:116616131:NM_014112:c.2065C>T) and was found to be a denovo mutation, which caused a change from Gln to the stop codon (chr8:116616131:NM_014112:c. 014112: c.2065C>T, p.Gln689*). After that, the translation is stopped, and the mutation results in a protein with a loss of function that is a truncated protein. The variation is not present in healthy people or in genetic databases. The frequency of the variant is not included in the HGMD, 1000Genomes, and ExAC databases, and the classification of the sequence variant according to the American College of Medical Genetics and Genomics(ACMG)guidelines: a Very Strong Evidence of Pathogenicity PVS1_Very Strong: When the pathogenicity mechanism of a gene is a loss-of-function (LOF), this sequence variant is classified as: a very strong evidence of pathogenicity PVS1_VeryStrong:indicates that the pathogenicity mechanism of a gene is either loss-of function (LOF) or nonsense (LOFs); alternatively, the shear (LD) and shear (TT) variations are changed at position 1 or 2, and the start codon variants are single or multiple exon deletion variants in the gene b Strong pathogenicity evidence PS2_Strong: Denovo variants with no family history (verified in the family lineage); c Supporting pathogenicity evidence PS4_Supporting: Variants that PS4_Supporting:variants occurring at a significantly higher frequency in the relevant patient population than in the control population; d Supporting pathogenicity evidence PM2_Supporting: variants that are not found (or have a very low frequency in the recessive mode of inheritance) in normal control populations in the ESP database, the 1,000 Genomes database, and the EXAC database; e Supporting pathogenicity evidence PP4: the clinical phenotype or family history of the variant's carriers is highly consistent with the characteristics of a single-gene genetic disease. Supports evidence of pathogenicity PP4: the clinical phenotype or family history of the variant carrier is highly consistent with the characteristics of a monogenic genetic disease.

Based on the ACMG guidelines, this variant has been classified as having two pieces of strong pathogenicity evidence and one piece of pathogenicity evidence 2 PS2_Strong+2 PM2_Supporting.This classification suggests that it may be a pathogenic variant. The Mutation Taster prediction was "likely pathogenic", and the phenotype of hair, eyebrow, bone changes and facial features of the patient was consistent with this monogenic disease. Combining the patient's clinical features, mutation test results and protein function prediction results, and the clinic, the diagnosis of TRPS was clear. The gene mutation was not reported after searching various databases, suggesting that it is a new mutation.


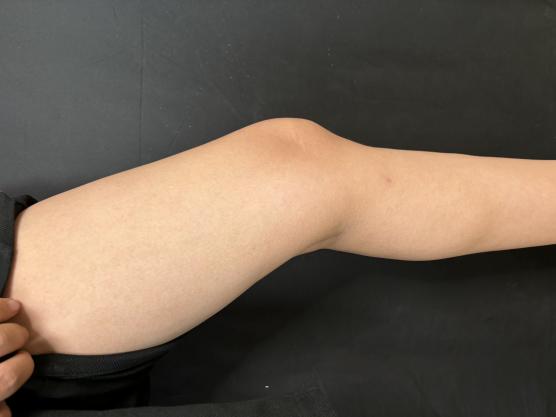

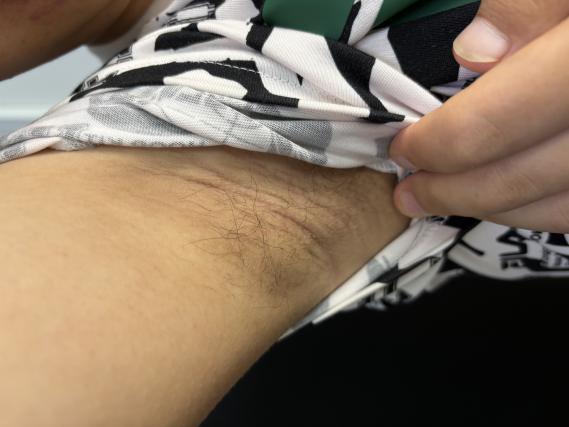

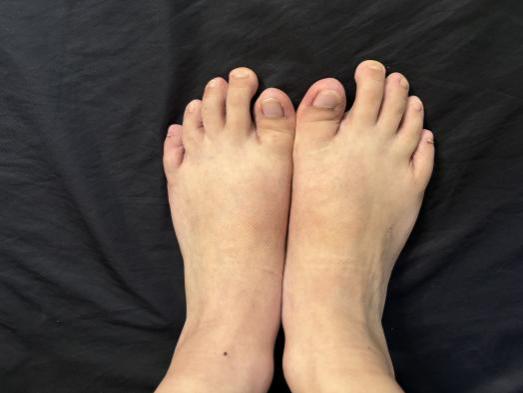

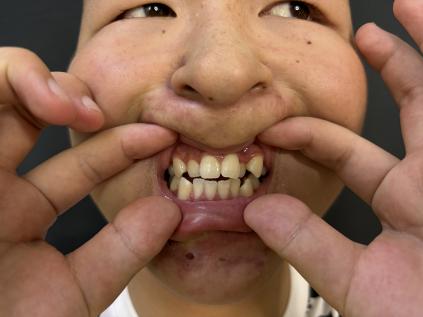

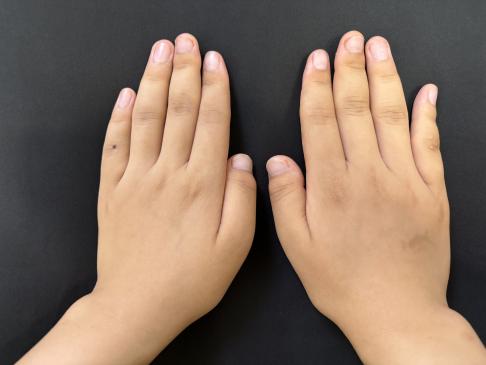

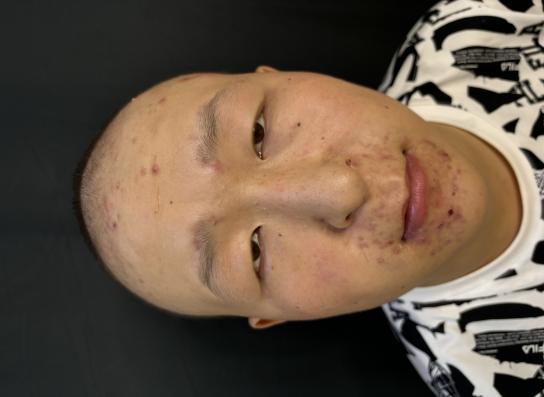

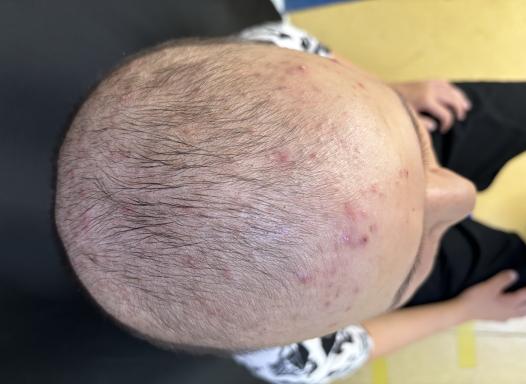


b

c

d

e

f

g

a

Figure 1 Clinical manifestations of the proband. a. diffuse sparse hair with varying thickness and length, soft and yellowish colour; b. special facial features include sparse outer eyebrows, fewer eyelashes, a bulbous nose, a medium length, a thinner upper lip, and multiple erythema, follicular papules, and pustules on the face, mouth area, and scalp; c and d. there is less hair on the whole body, and sparse hair on the armpits and limbs; e. uneven arrangement of teeth (supernumerary teeth, crowding); f and g. normal toenails. The thumb and toe are noticeably shorter.


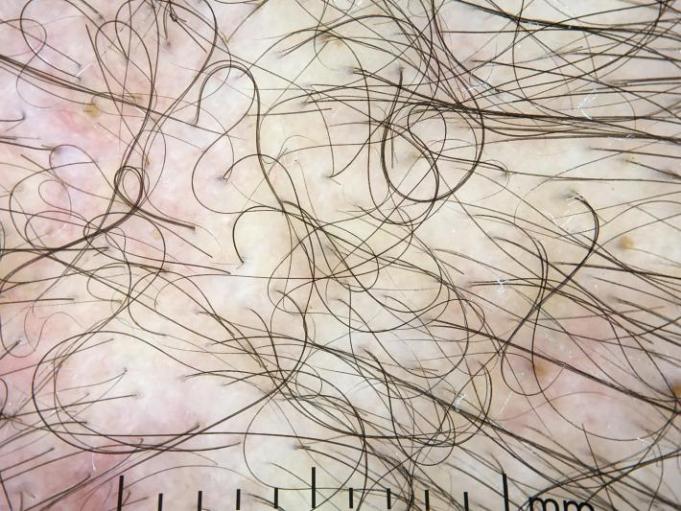

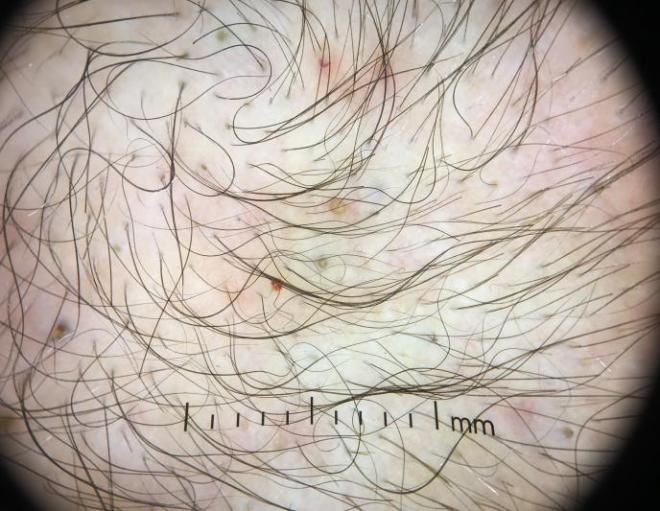


b

a

Figure 2 Dermoscopy detection results. The proportion of single hair follicle units has significantly increased, the diameter of the hair shaft has become thinner, and broken hair can be seen, with multiple follicular papules and pustules.


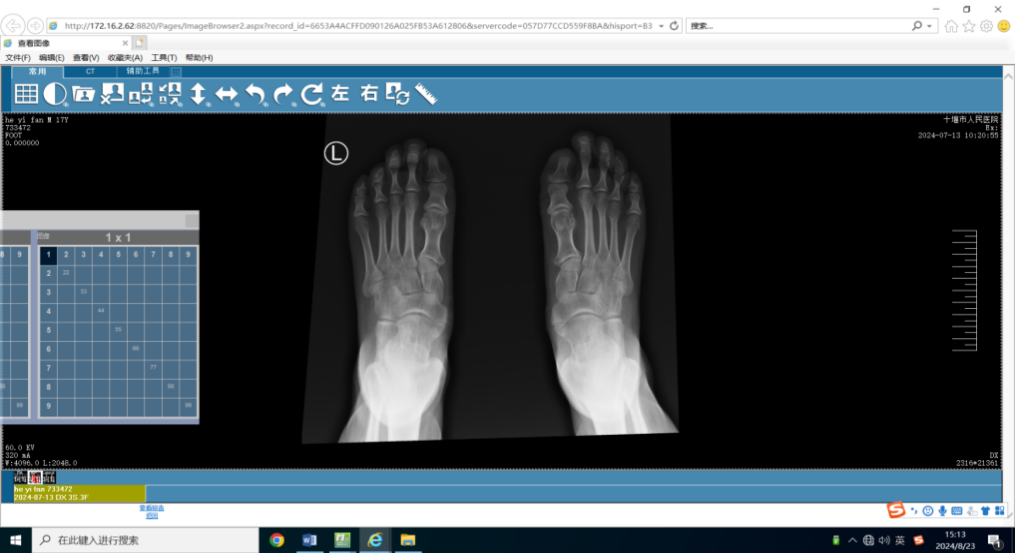

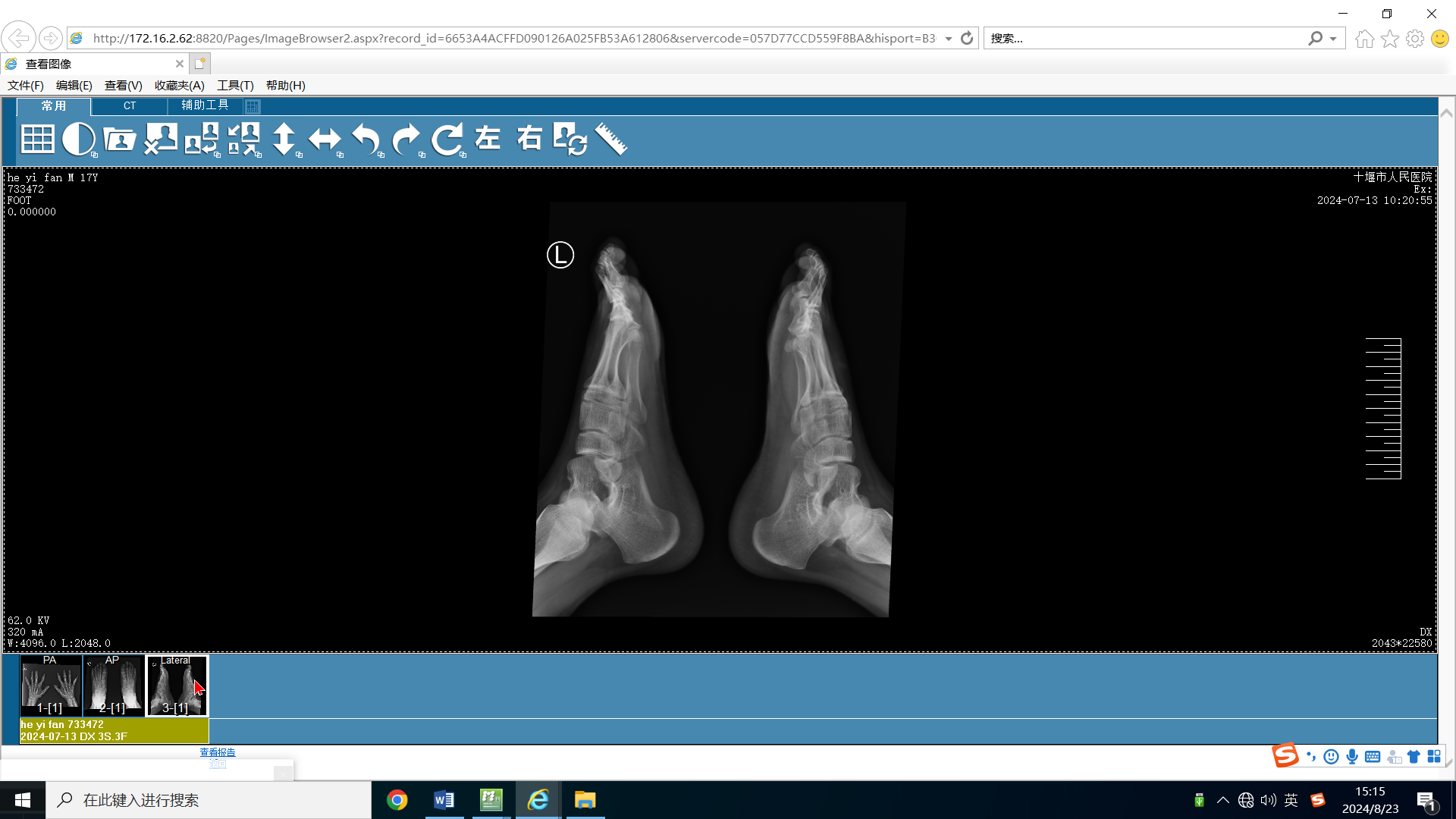

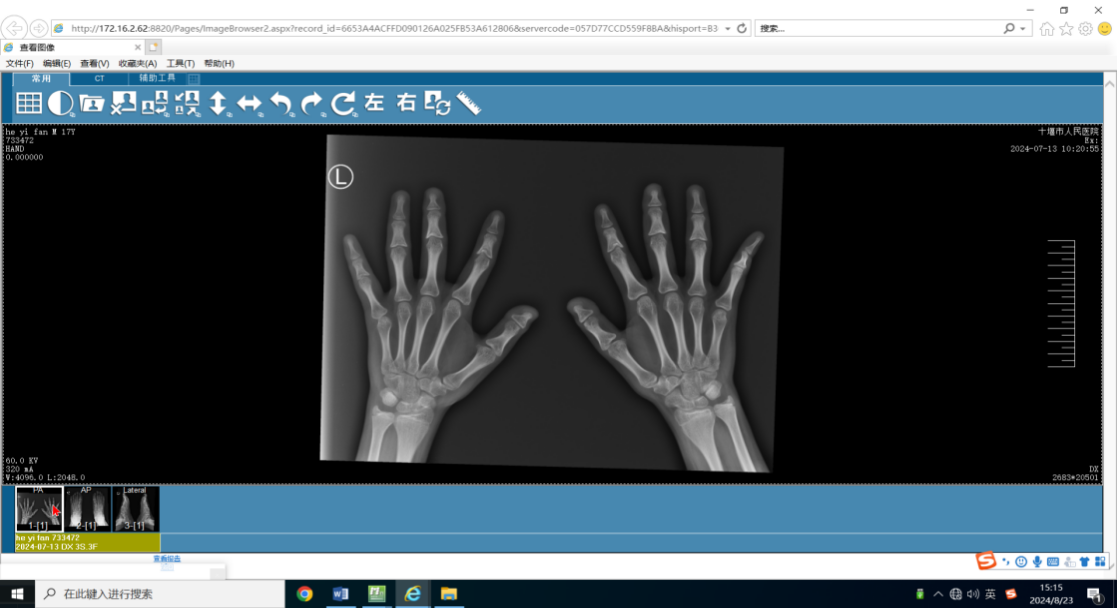


a

b

c

Figure 3 X-ray examination. a. Bilateral X-ray anteroposterior view: short proximal phalanges of the big toe of both feet; b. Bilateral X-ray lateral view; c. Bilateral X-ray: The distal phalanges of both thumbs are short, the base of the middle phalanges of the 2nd to 5th fingers is depressed, and the adjacent phalanges become pointed.

**The proband**

**Father**

**Mother**


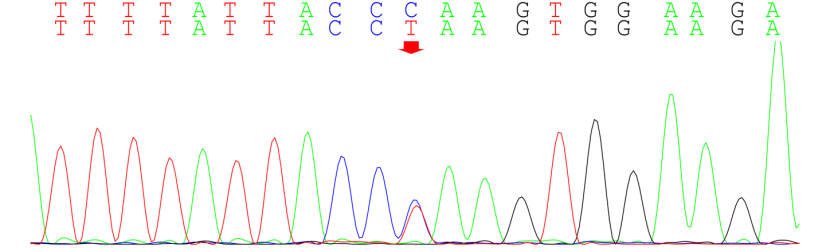

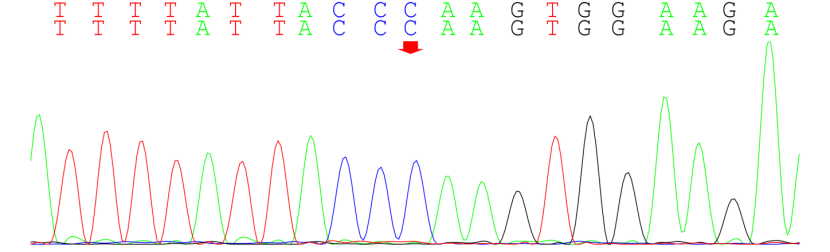

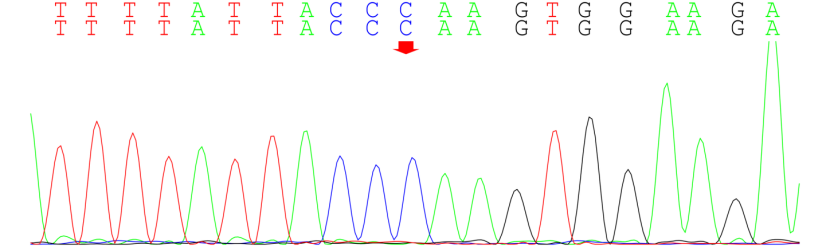


Figure 4: Family Sanger sequencing results. There is a heterozygous nonsense mutation on exon 4 of the TRPS I gene in the proband, c.2065C>T, p.Gln689 *. The proband's parents have no gene mutation at this locus.
